# Long-term use of interleukin-1 inhibitors reduce flare activity in patients with fibrodysplasia ossificans progressiva

**DOI:** 10.1093/rheumatology/keae255

**Published:** 2024-05-11

**Authors:** Ruby Haviv, Leonid Zeitlin, Veronica Moshe, Amit Ziv, Noa Rabinowicz, Fabrizio De Benedetti, Giusi Prencipe, Valentina Matteo, Carmen Laura De Cunto, Edward C Hsiao, Yosef Uziel

**Affiliations:** Pediatric Rheumatology Unit, Department of Pediatrics, Meir Medical Center, Kfar Saba, Israel; School of Medicine, Faculty of Medical and Health Sciences, Tel Aviv University, Tel Aviv, Israel; School of Medicine, Faculty of Medical and Health Sciences, Tel Aviv University, Tel Aviv, Israel; Pediatric Orthopedic Department, Dana-Dwek Children's Hospital, Sourasky Medical Center, Tel Aviv, Israel; Pediatric Rheumatology Unit, Department of Pediatrics, Meir Medical Center, Kfar Saba, Israel; Pediatric Rheumatology Unit, Department of Pediatrics, Meir Medical Center, Kfar Saba, Israel; School of Medicine, Faculty of Medical and Health Sciences, Tel Aviv University, Tel Aviv, Israel; Pediatric Rheumatology Unit, Department of Pediatrics, Meir Medical Center, Kfar Saba, Israel; Division of Rheumatology, Bambino Gesù Children’s Hospital IRCCS, Rome, Italy; Division of Rheumatology, Bambino Gesù Children’s Hospital IRCCS, Rome, Italy; Division of Rheumatology, Bambino Gesù Children’s Hospital IRCCS, Rome, Italy; Rheumatology Section, Department of Pediatrics, Hospital Italiano de Buenos Aires, Buenos Aires, Argentina; Division of Endocrinology and Metabolism, Institute for Human Genetics, The Eli and Edythe Broad Center of Regeneration Medicine and Stem Cell Research, Department of Medicine, University of California, San Francisco, USA; Pediatric Rheumatology Unit, Department of Pediatrics, Meir Medical Center, Kfar Saba, Israel; School of Medicine, Faculty of Medical and Health Sciences, Tel Aviv University, Tel Aviv, Israel

**Keywords:** fibrodysplasia ossificans progressiva, heterotopic ossification, interleukin, IL-1, IL-1β, anakinra, canakinumab

## Abstract

**Objectives:**

Fibrodysplasia ossificans progressiva (FOP) is one of the most catastrophic forms of genetic heterotopic ossification (HO). FOP is characterized by severe, progressive inflammatory flare-ups, that often lead to HO. The flare-ups are associated with increased inflammatory cytokine production, suggesting auto-inflammatory features driven by IL-1β. This study describes the short- and long-term responses of FOP patients to anti-IL-1 therapy.

**Methods:**

Previously, we reported that a patient with FOP treated with anti-IL-1 agents showed dramatically lower rates of flare-ups, improved flare-up symptoms, decreased use of glucocorticoids and apparently decreased size of residual lesions. Plasma analyses also showed marked elevation in IL-1β levels during a FOP flare, further supporting a role of IL-1β in the pathogenesis of FOP flares. Here, we report results from long-term therapy with IL-1 inhibitors in that patient and describe 3 additional patients, from two medical centres.

**Results:**

All 4 patients showed persistent improvement in flare activity during treatment with IL-1 inhibitors, with minimal formation of new HO sites. Two patients who stopped therapy experienced a resurgence of flare activity that was re-suppressed upon re-initiation. These patients had IL-1β levels comparable to those in IL-1β-driven diseases. Child Health Assessment Questionnaires confirmed extensive subjective improvements in the pain and general health visual analogue scales.

**Conclusion:**

This case series demonstrates significant benefits from IL-1 inhibitors for reducing flare activity and improving the general health of patients with FOP. These data provide strong support for additional studies to better understand the function of IL-1 inhibition, primarily in reducing the formation of new HO.

**Funding:**

RH received support from the International FOP Association ACT grant; ECH received support from NIH/NIAMS R01AR073015 and the UCSF Robert Kroc Chair in Connective Tissue and Rheumatic Diseases III.

Rheumatology key messages

- Patients with fibrodysplasia ossificans progressiva (FOP) have clinical features suggestive of auto-inflammatory flares that may be driven by IL-1 mediated pathways.
- IL-1 inhibitors appear to reduce the rate of and ameliorate FOP flares, may decrease the need for glucocorticoids during flares, and improve patient-reported well-being.
- Further studies should investigate whether reduced flare activity using IL-1 inhibitors preserves long-term function by limiting new HO.

## Introduction

Fibrodysplasia ossificans progressiva (FOP) is an ultra-rare genetic disease with an estimated prevalence of 1:1.2–1.5 million [1]. It is among the most catastrophic forms of heterotopic ossification (HO) [2] because of the progressive, irreversible accumulation of heterotopic bone and subsequent loss of mobility and independence. FOP is caused by activating mutations (mostly, the highly recurrent c.617G>A/p.R206H missense gain-of-function mutation) in the *ACVR1/ALK2* gene, that encodes the type 1 activin A receptor and leads to aberrant activation of the bone morphogenetic protein pathway and new neo-ligand activity of the activin A receptor [3].

Clinically, FOP is characterized by severe cumulative heterotopic bone formation, but bilateral deformities of the great toe can be seen at birth in about 80% of patients [4]. Additional skeletal deformities are known [5]. Patients often experience painful, indurated soft tissue swellings and masses that develop in response to even mild trauma and often progress to HO masses [2]. These flare-ups are occasionally associated with elevated inflammatory markers and sometimes respond to anti-inflammatory agents [6–9]. Flare-ups often begin during the first decade of life. By the third decade, most FOP patients are wheelchair-bound [2]. A major cause of morbidity is extensive joint ankylosis. This includes the temporomandibular joints, with nutritional compromise; the intercostal joints and rib cage, with thoracic insufficiency syndrome and restricted respiratory function; and limb joints, with loss of ambulation [2]. HO at non-joint sites like the buttocks and back also prevent some patients from sitting comfortably or lead to pressure ulcers. Average life expectancy is about 50 years [2, 10].

Currently, only limited clinically effective disease-modifying treatments for FOP are available. Standard therapies include anti-inflammatory agents, such as anti-leukotrienes, glucocorticoids, non-steroidal anti-inflammatory drugs and mast-cell stabilizers. Palovarotene was approved by Health Canada (https://canjhealthtechnol.ca/index.php/cjht/article/view/SR0761/1400) and by the United States Food and Drug Administration (https://www.fda.gov/drugs/news-events-human-drugs/fda-approves-first-treatment-fibrodysplasia-ossificans-progressiva) to reduce HO in females ages 8 years and older and males 10 years and older, diagnosed with FOP. However, the prevention of flare-ups and new HO sites remains inadequately managed [4].

FOP HO flare-ups occur via the bone morphogenetic protein pathway [8, 9] and share a striking resemblance to flares in well-characterized auto-inflammatory diseases, typified by innate immune cell activation, elicited by trivial triggers that usually do not cause a response in healthy individuals. The inflammatory response in these diseases is associated with overproduction of innate immune cytokines. IL-1β has been shown to have important roles in causing tissue damage, in some patients [11]. Accordingly, increasing evidence supports a major role for inflammation in HO [8, 9, 12–14]. Elevated pro-inflammatory cytokines and increased pro-inflammatory monocyte subsets have also been found in the blood of FOP patients [6]. Consistent with these observations, serum levels of TNF-α and IL-1β were significantly increased within 48 h post-injury, in a mouse model of trauma-induced HO [8].

There is an increased incidence of flare-ups in younger FOP patients, mostly below the age of 15 years [2], which indicates higher disease activity levels during childhood. The progressive, cumulative nature of HO in FOP indicates that interventions in early childhood are critical to reduce the lifelong, cumulative consequences of HO in FOP.

Therapies targeting IL-1, such as anakinra (IL-1 receptor antagonist), or canakinumab (anti-IL-1β monoclonal antibody) have extensive paediatric experience [11]. We previously reported that treatment with anakinra or canakinumab significantly reduced FOP flare-ups in a patient. This patient also demonstrated increased responsiveness to glucocorticoids used to manage flare-ups [15].

Here, we report our long-term experience with IL-1 inhibitors for preventing FOP flare-ups and managing FOP in 4 patients from 2 medical centres.

## Methods

Patients were diagnosed based on typical clinical features of FOP and carried the c.617G>A/p.R206H mutation. All had severe, active disease, with paroxysmal HO flares, despite standard-of-care therapy. Flare-up activity was based on patients’ and parents' reports and diaries, according to symptoms, including pain, swelling, erythema, induration and acute loss of motion. In addition, these were verified by the physicians through physical examinations in all cases and imaging tests, when performed. These were all documented in the medical records, with times in relation to the reports from the patients and their families. Parents of patients treated with IL-1 inhibition therapy completed the Child Health Assessment Questionnaire (CHAQ) questionnaire to better understand their functional status, after being on IL-1 inhibition therapy for over 1 year. We focused on changes in the pain and general health visual analogue scales (VAS) sections.

### Cytokine measurements

Blood samples were obtained during flare activity or routine follow-up while under biologic treatment. Plasma IL-1β levels were measured using the Human IL-1β/IL-1F2 Quantikine High Sensitivity ELISA Kit (HSLB00D, R&D Systems, Inc., Minneapolis, MN).

### Statistics

Flare frequency was determined by the number of documented flare-ups over the treatment period and expressed as flare-ups/month. Flare frequency for the pre-IL-1 inhibitor, IL-1 inhibitor pauses and during IL-1 therapy phases, were combined and averaged. Statistical significance for anti-IL-1 treatment *vs* non-treatment phases was determined using a two-tailed paired *t*-test (Excel, Microsoft Corporation).

### Role of the funding source

The funding sources had no role in the writing of the manuscript or in the decision to submit it for publication.

### Case presentations

Three female and 1 male patient with FOP, diagnosed clinically (toe malformations, FOP-like indurated flares and generalized HO) and genetically (ACVR1^R206H^ mutation) and higher than average flare activity [16], were treated with IL-1 inhibitors. IL-1 inhibition onset ranged from 23 months to 15 years (Table 1). All received chronic standard-of-care treatment [4], plus palovarotene [17] (patients 2 and 4), intermittent pamidronate infusions (patient 1), sirolimus (patient 2), imatinib [18] (patient 4) or tranexamic acid (patient 3). Nonetheless, all experienced persistent flare activity before IL-1 inhibitors were initiated.

**Table 1.** Summary of flare-up rates of four patients with FOP treated with anti-IL-1 therapy

Patient	Age at diagnosis	Flare rate before initiating IL-1 inhibitors	Age	IL-1 inhibitor and dosage	Flare rate (#flares/total months) during treatment with IL-1 inhibitors, important notes	Flare reduction rate (%)
1. Male^a^^,^^c^	13.5 years	40 flares/13 months = 3.07/month	15 years	ANA 100 mg daily	3/4 = 0.75/month	89%
			15 years, 4 months	CKB 300 mg monthly	5/4 = 1.25/month	
			15 years, 8 months	CKB Paused	5/1 = 5/month^d^	
			15 years, 10 months	CKB 150 mg twice monthly	13/16 = 0.81/month	
			17 years, 2 months	Restart, split CKB dose ∼ 2 mg/kg (up to 250 mg) twice monthly	8/23 = 0.35/month^e^	
			Weighted flare rate with IL-1 inhibitors		0.62/month	
2. Female^a^	3 years	23 flares/18 months = 1.28/month	5 years, 7 months	CKB ∼ 4 mg/kg monthly	0/2 = 0/month	80%
			5 years, 9 months	CKB Paused	3/1 = 3/month^f^	
			5 years, 11 months	Restart CKB ∼ 4 mg/kg monthly	9/34 = 0.26/month^g^	
			Weighted flare rate with IL-1 inhibitors		0.25/month	
3. Female^a^	16 months	4 flares/4 months = 1/month	23 months	CKB ∼ 4 mg/kg monthly	4/23 = 0.17/month^h^	83%
4. Female^b^	3 years	8 flares/14 months = 0.57/month before imatinib; 9/13 = 0.69/month before ANA	14 years, 3 months	ANA 100 mg daily	5/18 = 0.27/month^i^	61%
			14 years, 5 months	CKB 3.5–6 mg/kg/m		

a Meir Medical Center, Israel, affiliated with the School of Medicine, Faculty of Medical and Health Sciences, Tel Aviv University, Tel Aviv University, Tel Aviv, Israel.

b University of California, San Francisco, CA, USA.

c Patient 1 was described previously [15].

d New HO sites developed.

e At least 6/8 (75%) were at existing sites.

f New HO sites developed.

g 4/9 (44%) were not treated with steroids. At least 6/9 (67%) were at known sites and 4 (67%) of these were at a surgical site.

h All were around existing masses, were partially treated with glucocorticoids and decreased in size over time.

i Patient reported decreased pain and induration at flare sites.

ANA: anakinra; CKB: canakinumab.

**Figure 1.**
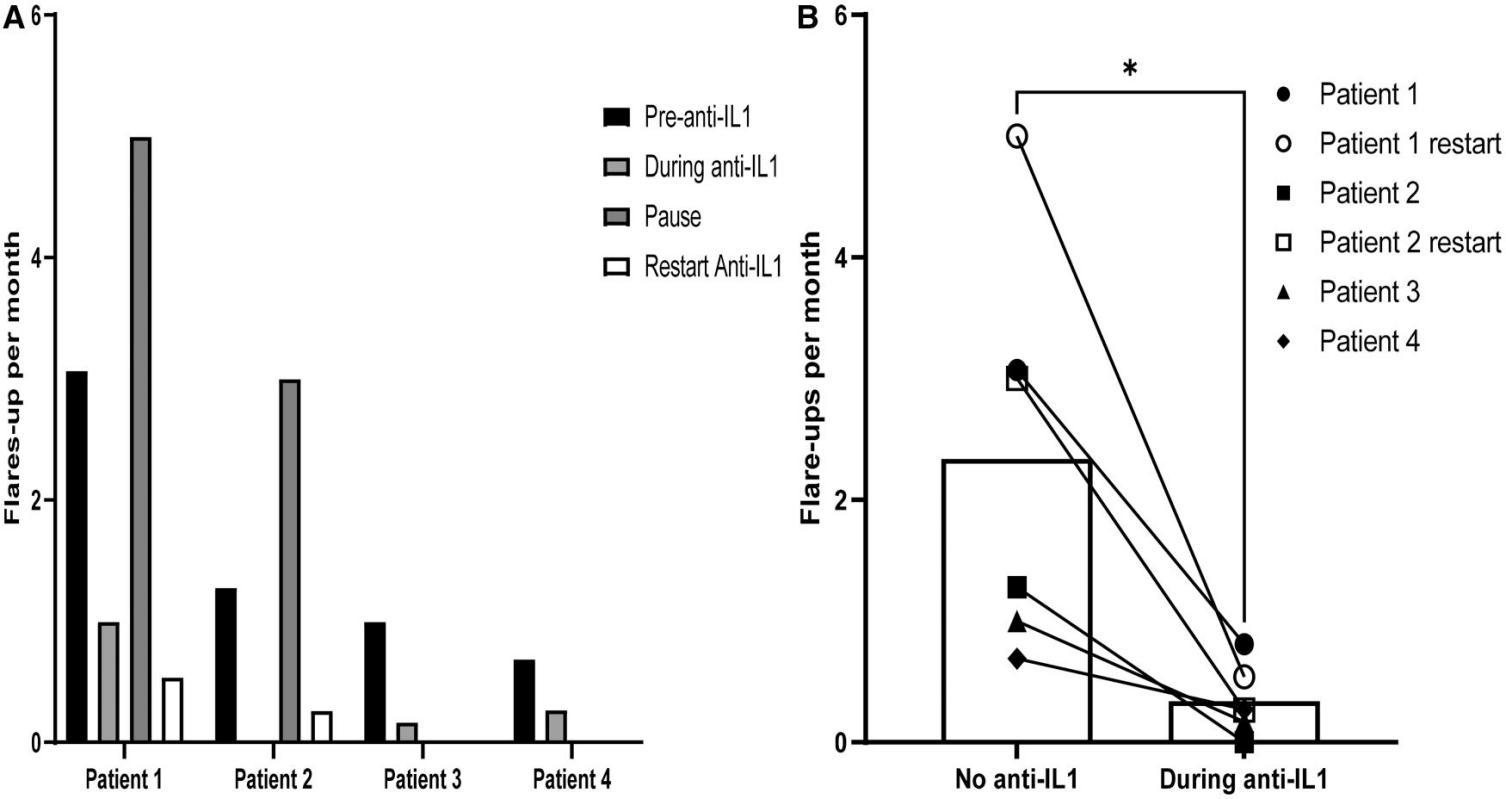
Flare rates of patients with FOP treated with anti-IL-1 therapy. (A) Individual patient flare-up rates per month before initiation of therapy, during therapy, during a pause and after restarting it. (B) Average flare-up rates/month for all treatment phases without anti-IL-1 *vs* with anti-IL-1. *P < 0.01

After commencing IL-1 inhibitors, flare rates decreased ∼76%, from a mean of 1.51–0.36 flares/month (*P* < 0.05). Following the treating physicians’ decisions, Patients 1 and 2 stopped canakinumab for 7.5 weeks. Due to HO flare-ups recurring at similar rates to pre-IL-1-inhibition, the treatment was reinitiated (Table 1, Fig. 1A, B). The combined data from these periods yielded a decreased flare rate of 82.5%, from a mean of 1.65–0.29 flares/month (*P* < 0.03). Most flare-up sites during the follow-up period, while the patients were treated with IL-1 inhibitors, were in the axial and not the appendicular skeleton and almost all were known HO sites before initiation of IL-1 inhibition (most were augmentations of existing axial skeleton HO sites; most prominent in the neck, scapulae and trapezius).

The patients' parents completed the CHAQ. Extensive improvements in the pain and general health visual analogue scales (VAS) sections for 3 of the 4 FOP patients were noted: 54% decrease in pain and 48% increase in general health. The individual changes per patient are detailed in Table 2. The CHAQ VAS results for patient 4 are also shown in the table, but change could not be measured, as only the post-treatment questionnaire was completed.

**Table 2.** Changes in the pain and general health visual analogue scales sections of the Child Health Assessment Questionnaire

Patient	Pain VAS (0 is ‘no pain’)			General health VAS (10 is ‘very good’)		
	Before initiating IL-1 inhibitors	During CKB treatment	Percent change	Before initiating IL-1 inhibitors	During CKB treatment	Percent change
1. Male^a^^,^^c^	8.6	5.0	−42%	4.7	7.1	+51%
2. Female^a^	5.8	2.2	−62%	2.1	7.9	+276%
3. Female^a^	1.3	0	−100%	6.1	10	+64%
Average (patients 1–3)	5.2	2.4	−54%	4.3	8.3	+48%
4. Female^b^	–	3.0	–	–	8.8	–

a Meir Medical Center, Israel, affiliated with the Faculty of Medicine, Tel Aviv University.

b University of California, San Francisco.

c Patient 1 was described previously [15].

CKB: canakinumab.

**Figure 2.**
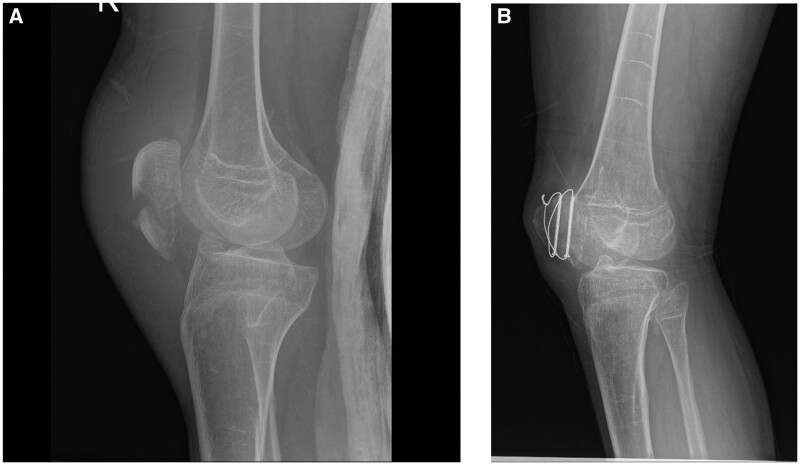
Images of the right knee and surrounding area of patient 1, before the surgical repair of his right patella (A) and 3.5 months after (B), with no radiological signs of heterotopic ossification

Patients 1 and 2 underwent surgery while on IL-1 inhibition therapy. In combination with high-dose methylprednisolone (30 mg/kg) and canakinumab therapy (4 mg/kg), no HO flare-ups were documented during and immediately post-surgery. Patient 1 underwent surgical repair of a fractured right patella in February 2022 (Fig. 2A). No new HO sites have been documented since, except one lump under the cast, which regressed quickly, leaving no radiographic signs 3.5 months after surgery (Fig. 2B). Patient 2 underwent two surgical procedures to extract heterotopic cervical bones (right sternocleidomastoid muscle site), that had caused extra-articular temporomandibular joint ankyloses. CT scans were performed before each operation [19] and once in between; no new HO sites were identified. More importantly, no HO flare-ups were documented during and immediately post-surgery, under combination therapy with methylprednisolone and canakinumab. However, the surgical site in patient 2 re-ossified within 3–4 months. Through January 2024, neither patient has developed a new HO site since starting IL-1 inhibitory treatment: 6 years for patient 1 and 4.5 years for patient 2. Through January 2024, the only adverse events were transient mild pain around injection sites (patient 4) and mild thrombocytopenia (>110 000 platelets/mm^3^) that might be related to canakinumab in patient 1. This has been stable since January 2022 and improved after canakinumab was decreased to ∼4 mg/kg/month. Patient 1 improved his diet and began supervised exercise activity. His BMI decreased from 34 to 24 over 20 months.

### Plasma IL-1β levels in patients 1 and 2

**Figure 3.**
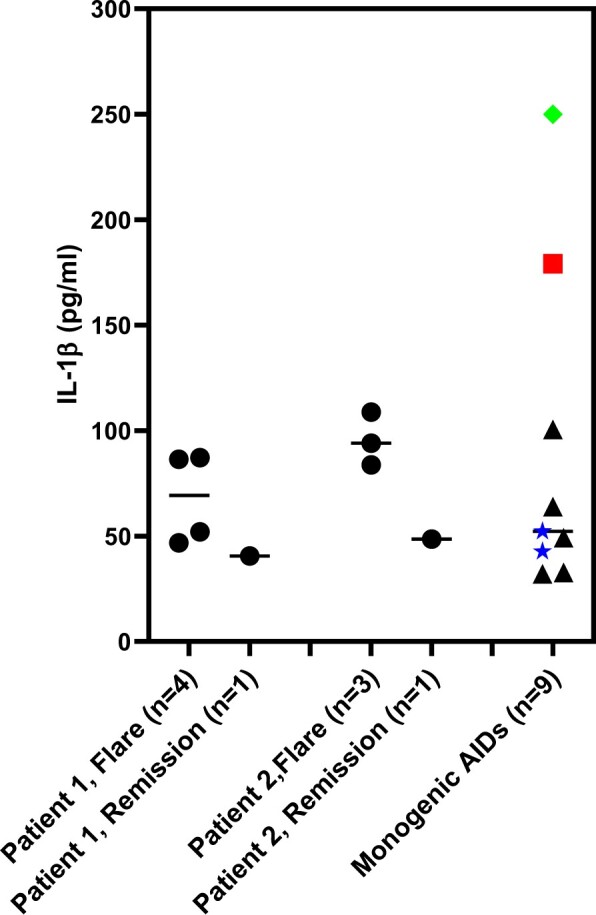
IL-1β plasma levels in FOP Patients 1 and 2 on canakinumab treatment. IL-1β levels were measured by ELISA in plasma samples collected during treatment with canakinumab from patient 1 (four samples during flares and one during remission) and patient 2 (three samples during flares and one during remission). IL-1β levels were also measured in plasma samples from patients with IL-1-driven monogenic auto-inflammatory diseases, including cryopyrin-associated periodic syndrome (*n* = 5, triangles), FMF (*n* = 1, square), TNF-associated periodic syndrome (*n* = 2, asterisks) and mevalonate kinase deficiency (*n* = 1, diamond) who were undergoing treatment with canakinumab

We evaluated the levels of IL-1β in plasma samples collected from patients 1 and 2 with ELISA. In all plasma samples collected before initiation of treatment with canakinumab, IL-1β levels were undetectable (<0.1 pg/ml, data not shown). In contrast, high levels of IL-1β were measurable in all samples collected from FOP patients while receiving canakinumab. This finding is consistent with observations in patients with cryopyrin-associated periodic syndromes (CAPS) [20], FMF, mevalonate kinase deficiency (MKD) and TNF-associated periodic syndrome (TRAPS) [21]. In these diseases, in which the pathogenic role of Il-1β is proven and canakinumab efficacy demonstrated [22, 23], the detectable levels of IL-1β are due to the formation of canakinumab/IL-1β complexes allowing the detection of IL-1β produced *in vivo* [20]. The IL-1β levels detected in FOP patients were comparable to those observed in patients with monogenic autoinflammatory diseases driven by IL-1, such as CAPS, FMF, MKD and TRAPS, under treatment with canakinumab (Fig. 3). In patients 1 and 2, a trend towards higher IL-1β levels during flares than during the remission phases of the disease was observed (Fig. 3). As expected and consistent with data obtained from untreated FOP patients, IL-1β levels were also undetectable in plasma samples from patients with CAPS, FMF, MKD or TRAPS who were not undergoing treatment with canakinumab (data not shown).

## Discussion

Our experience with IL-1 inhibitors for paediatric FOP patients suggests that they are very effective and well-tolerated, even after long-term treatment. Anakinra and particularly canakinumab, appear to dramatically ameliorate painful and persistent inflammatory flare-ups. Flare rates decreased dramatically by about 82%. All 4 patients demonstrated significant improvement and disease stability, with no major adverse reactions to the IL-1 inhibitors. Axial HO flare-ups were documented, almost all within existing sites of HO masses, but at a significantly lower rate. Temporary cessation of canakinumab was associated with increased flare activity (similar to the flare rate documented prior to IL-1 inhibition), which was suppressed upon reinitiation.

Although paediatric intervention is likely to have the highest impact on the prognosis of this devastating disease, there are currently no established disease-modifying treatments for FOP [2]. Off-label medications have been tried, including imatinib mesylate (patient 4), a selective tyrosine kinase inhibitor, that appears to be well-tolerated and helps decrease the intensity of HO flares [18]. Tumour necrosis factor-α inhibitors were not successful (experience of ECH and personal communication to RH). When the flare-up lumps appear, high-dose oral or intravenous glucocorticoids, possibly combined with bisphosphonate remain the standard of care [4], but are not recommended and are probably ineffective for prophylaxis (see data from Patients 1 and 4). Sirolimus (rapamycin), an immunosuppressant that inhibits mTOR1 kinase activity, decreased HO in FOP mouse models and inhibited the pathway in FOP-induced pluripotent stem cell models [24]. Patient 2 did not respond significantly to sirolimus during a short trial. A phase 2 clinical trial using rapamycin is ongoing in Japan (https://center6.umin.ac.jp/cgi-open-bin/ctr/ctr_view.cgi?recptno=R000032495). Investigational agents in clinical trials include saracatinib (AZD0530), an ID-1 inhibitor (ClinicalTrials.gov NCT04307953) [25], garetosmab, an anti-activin A antibody (ClinicalTrials.gov NCT05394116) [26]; and palovarotene, a retinoic acid receptor-γ agonist (ClinicalTrials.gov NCT02279095 and NCT03312634) [17]. However, some young patients receiving palovarotene developed early epiphyseal closure and palovarotene (and other retinoids) are known teratogens and may also induce pancreatitis, hearing and vision impairment, mouth ulcers, sensitivity to sunlight and retinoid skin reactions. Identifying paediatric therapies with known potential side effects and tolerability are essential for managing FOP.

Previous data linked FOP to increased pro-inflammatory cytokine production, including IL-1 [6, 7, 15, 19]. A very recent study reported a significant decrease in flare activity with tofacitinib treatment in 13 children with genetically-confirmed FOP [27]. These findings are similar to our observations with IL-1 inhibitory therapy. IL-1 receptor antagonist levels (IL-1RA) were decreased under tofacitinib treatment in 4 of the 5 patients with FOP, whose IL-1RA levels were measured in this study [27]. IL-1RA is secreted in parallel with IL-1β and its levels have already been shown to return to normal ranges, along with IL-6, C-reactive protein and serum amyloid A, under canakinumab treatment [20]. Tofacitinib, a Janus kinase (JAK) inhibitor, inhibits granulocyte macrophage colony-stimulating factor (GM-CSF)-induced JAK2-mediated signal transduction and abrogates GM-CSF-induced IL-1β and cleaved caspase-1 secretion from neutrophils. It also reduces IL-1β secretion by abrogating NLR family pyrin domain-containing 3 (NLRP3) inflammasome expression [28]. Tofacitinib was also beneficial in a case series of patients diagnosed with FMF, a well-known, IL-1-driven disease [29].

In the present study, high IL-1β levels were found in FOP patients treated with canakinumab, particularly during flares. Although this seems paradoxical, due to the very short 2.5 h half-life of IL-1β, low/undetectable levels are found in patients with inflammatory diseases, even when increased IL-1β production is pathogenic. In patients with CAPS, FMF, MKD and TRAPS, all diseases in which the pathogenic role of IL-1β is proven and canakinumab efficacy demonstrated [22, 23]. IL-1β was detectable after initiation of canakinumab, due to the formation of an IL-1β/canakinumab complex with subsequent stabilization of the circulating cytokine. In our patients with FOP, after initiation of canakinumab, we consistently found detectable amounts of IL-1β. IL-1β levels were comparable to those observed in canakinumab-treated patients with monogenic auto-inflammatory diseases driven by IL-1β, such as CAPS, FMF, MKD and TRAPS. Moreover, IL-1β levels were higher during flares than remissions. These results demonstrate increased IL-1β production in FOP and further support its role in disease pathogenesis; thereby, adding to the rationale for using IL-1 inhibitors to treat FOP.

This report has a few limitations. Since the formation of heterotopic bony masses is not always preceded by a typical ‘flare-up’ and since the HO burden increases with time [2], radiological changes in the HO burden and formal changes in functionality, such as those reported with the Cumulative Joint Analogue Involvement Scale [30] (which were not assessed here) may have provided clearer, objective data regarding the patients’ status. However, the subjective improvements in function and pain confirmed by the CHAQ questionnaire (Table 2) and the decrease in inflammatory symptoms of flares are significant advances for these difficult-to-manage cases. Moreover, reductions in flare rate and the extent of site involvement of the flares achieved during treatment with IL-1 inhibitors in younger patients, may result in a cumulative reduction in HO burden as patients age. This important pathophysiological link remains to be elucidated.

Although anakinra and canakinumab appeared to reduce flare-ups significantly, optimal dosing regimens are undefined. The patients reported here had intractable flares that failed to respond to standard-of-care management and received rescue therapy with IL-1 inhibitors. It is unknown whether IL-1 inhibitors will benefit patients with fewer flares or adults with FOP. The observations that Patients 1 and 2 did not experience *new* sites of HO after surgery are promising. However, the finding that HO recurred at an existing site after surgery suggests that further study is needed before IL-1 inhibitors could be considered for prophylactic use in patients with FOP undergoing surgery.

## Conclusions

Growing evidence indicates that the nature of flare-ups in FOP is inflammatory and that anti-inflammatory approaches provide critical therapeutic strategies to disrupt this process. Experimental data and our findings in this observational clinical case series suggest that IL-1 has a role in the formation of HO in FOP. The natural history of FOP, based on ∼13% of diagnosed FOP patients worldwide [2], indicates higher disease activity at younger ages. It is therefore logical to extrapolate that IL-1 inhibition may be more pronounced before adulthood. IL-1 inhibitors are expensive and recently, the JAK inhibitor tofacitinib was shown to be beneficial for reducing flares in 13 patients with FOP [27]. Although the current report provides further support to the ‘anti-inflammatory approach’, it is important to emphasize that JAK inhibitors are related to a wide range of side effects due to their broad mechanism of action. Paediatric experience with this group of relatively new agents is currently limited. Thus, additional studies are needed to determine the contribution of IL-1 compared with other inflammatory cascades and mediators.

The four cases presented here demonstrated clear clinical benefits from IL-1 inhibitors for patients with FOP, particularly with a marked decrease in flare frequency and new lumps, decreased pain and glucocorticoid use and improvement in patient-reported well-being. In addition, the minimal HO formation after surgery in two of the patients receiving IL-1 inhibitors, suggests that IL-1 may have wide-ranging impact for patients with FOP. Thus, IL-1 inhibition may be a potential strategy for managing FOP providing hope for improved quality of life to these patients. These results support the need for further studies to understand the impact of IL-1 inhibition on new HO formation and functionality.

## Data Availability

The data that support the findings of this study are available from the corresponding author upon reasonable request.
